# Feasibility of an Assessment Tool as a Data-Driven Approach to Reducing Racial Bias in Biomedical Publications

**DOI:** 10.1007/s10916-021-01777-w

**Published:** 2021-12-17

**Authors:** Siobhan Wescott, Ronn Johnson, Sangeeta Lamba, Devon Olson, Yolanda Haywood, Carolyn C Meltzer, Ricardo Correa

**Affiliations:** 1 College of Public Health, University of Nebraska Medical Center, 984365 Nebraska Medical Center, Omaha, NE US; 2 Creighton University School of Medicine, Omaha, NE US; 3 Rutgers Biomedical and Health Sciences, New Brunswick, NJ US; 4 University of North Dakota School of Medicine and Health Sciences, Grand Forks, ND US; 5 The George Washington University School of Medicine and Health Sciences, Washington, US; 6 Emory University School of Medicine, Atlanta, GA US; 7 University of Arizona College of Medicine, Phoenix, AZ US

**Keywords:** Racial Bias, Biomedical Literature, Framework, Editorial Policies

## Abstract

The editorial independence of biomedical journals allows flexibility to meet a wide range of research interests. However, it also is a barrier for coordination between journals to solve challenging issues such as racial bias in the scientific literature. A standardized tool to screen for racial bias could prevent the publication of racially biased papers. Biomedical journals would maintain editorial autonomy while still allowing comparable data to be collected and analyzed across journals. A racially diverse research team carried out a three-phase study to generate and test a racial bias assessment tool for biomedical research. Phase 1, an in-depth, structured literature search to identify recommendations, found near complete agreement in the literature on addressing race in biomedical research. Phase 2, construction of a framework from those recommendations, provides the major innovation of this paper. The framework includes three dimensions of race: 1) context, 2) tone and terminology, and 3) analysis, which are the basis for the Race Equity Vetting Instrument for Editorial Workflow (REVIEW) tool. Phase 3, pilot testing the assessment tool, showed that the REVIEW tool was effective at flagging multiple concerns in widely criticized articles. This study demonstrates the feasibility of the proposed REVIEW tool to reduce racial bias in research. Next steps include testing this tool on a broader sample of biomedical research to determine how the tool performs on more subtle examples of racial bias.

**Supplementary information:**

The online version contains supplementary material available at 10.1007/s10916-021-01777-w.

## Introduction

Biomedical journals have editorial independence. This provides room for a wide spectrum of research interests. However, the resulting disseminated nature of biomedical literature creates an obstacle to addressing difficult, systemic issues such as racial bias.

During the COVID-19 pandemic, the polarizing nature of race has come into sharp focus for the United States and its research community. A series of recent incidents, involving high impact factor journals, have drawn attention to the extent of those in research who lack a fundamental understanding of systemic racism [1]. Some of the recent editorials on addressing structural racism in medicine and biomedical publishing have been highly informative [2–4].

Many organizations, including scientific and medical professional societies and biomedical journals have made statements condemning health inequities. Khazanchi et al. [5] call on organized medicine to go beyond declaratory advocacy towards action. *They specifically recommend that research journals adhere to rigorous standards when publishing scholarly work on race, racial health disparities, and racism.*

A standardized assessment tool for racial bias would be an ideal method to operationalize recommendations on race in the biomedical literature. If such a tool were feasible, journals could flag manuscripts with the potential to contribute to the persistence of systemic racism in the scientific literature and in medicine [5] in the pre-publication phase.

Further, data generated from the use of a racial bias assessment tool could be compared across journals. Editorial boards, authors, and other key stakeholders could study the data to inform updates in the editorial process and/or education efforts that would reduce the likelihood of racial bias in the biomedical literature.

Because this is a highly sensitive topic, this article’s research team has representation from diverse voices. These include major categories of race in the US (African American, Latino/Latintx/Hispanic or LHS + , American Indian/Alaska Native, Asian, White), geographic areas (East Coast, South, Midwest, West), multiple institutions, and with collectively over a century of experience working on justice, diversity, equity and inclusion (including four Deans).

This article identifies (Phase 1), constructs (Phase 2), and then pilots (Phase 3) an assessment tool for the dimensions of race that could be incorporated in the editorial review process of biomedical journals.

## Phase 1: Identify recommendations in the literature

Led by a Masters level medical librarian (author DO), we used concepts for “racism, “medicine”, and “publication” to guide our structured literature review. The search terms used are identified in Table 1. The search was most recently conducted in PubMed on March 2021. Manual searches were conducted of references within resulting articles as well as in the following journals: *Racial and Ethnic Health Disparities, Social Science and Medicine,* and *Ethnicity and Health.*

**Table 1:** Literature Search

	Source	Search string
Database search	PubMed	("Editorial Policies"[Mesh] OR “Publications"[Mesh] OR "Publishing"[Mesh] OR “Terminology as Topic”[MAJR] OR "Periodicals as Topic"[MAJR]) AND ("Racism"[Mesh])
	Google Scholar	(race OR racism) AND (medical OR medicine OR genetic) AND (article OR literature OR publication OR publish)
Hand Search	Racial and Ethnic Health Disparities	racism
	Social Science and Medicine	racism
	Ethnicity and Health	racism

**Fig. 1:**
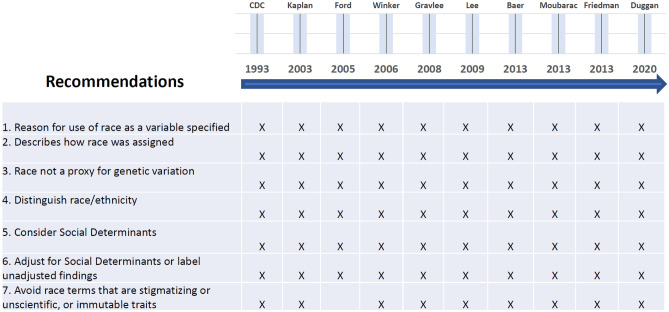
Assessing Agreement on Racially Responsive Recommendations from the Literature. Findings from the structured literature search are shown. Over a 27-year span, 10 articles [6–15] were found which had a similar set of recommendations about how to standardize the reporting of race in the biomedical literature and reducing racial bias

The findings from the literature search are summarized in Fig. 1. We identified 10 articles with clear recommendations for policy components, dating back to 1993 [6]. In the 27-year span of these articles, researchers have consistently drawn attention to the ambiguous, confusing, and, at times overtly racist, treatment of race in health research journal publications.

Race in research is a global problem. We would have liked to have had a worldwide approach in this paper. However, early in the process we surmised that race in the US has such a unique and high degree of complexity for biomedical health that an international approach is not yet feasible [6].

In 1993, the Centers for Disease Control and Prevention (CDC) and the Agency for Toxic Substances and Disease Registry sponsored a 2-day workshop to address the role of race and ethnicity in US public health surveillance. The workshop recommendations were published to highlight issues and outline key concepts, measures, and uses of race and ethnicity in public health surveillance along with practical strategies for improvement [6]. The report highlighted the impact of the US Office of Management and Budget's (OMB) Directive 15, “Race and Ethnic Standards for Federal Statistics and Administrative Reporting” on race data. The Directive was developed in 1977 to ensure the collection and use of compatible, nonduplicated, exchangeable racial and ethnic data by Federal agencies. It directs federal agencies to collect data on at least four racial groups: White, Black, American Indian and Alaskan Native, and Asian/Pacific Islander; and one ethnic group, Hispanic.

However, the 1978 OMB Directive 15 *explicitly notes the absence of scientific considerations* in the designation of these categories of race and ethnicity: “These classifications should not be interpreted as being scientific or anthropological in nature.” The report recommended use of self-identification of race and ethnicity, conceptualized a periodic review of definitions and uses of race and ethnicity, while clearly stating that because race and ethnicity are imperfect predictors of health status, information should be collected on other variables that would add a dimension of predictive power (e.g., formal years of education). The report also called for the need to clearly identify the reason for use of race data (e.g., to recognize health disparities), use context and potential intervening variables such as socio-economic status to analyze and report results, and to explicitly define the approach to measurement and the limitations of the race and ethnicity data [6, 8].

Ten years later, Kaplan & Bennett echoed and detailed further suggestions to curtail racism in research [7]. They called on article authors to contextualize any use of race, disambiguate race from ethnicity, always explain how participants’ races were derived or assigned, consider culturally relevant factors like socioeconomic status, and to cite evidence from gene studies when discussing genetic differences, among other preferred practices. Kaplan stated, “In describing racial/ethnic groups, authors should use terminology that is not stigmatizing, does not reflect unscientific classification systems, and does not imply that race/ethnicity is an inherent, immutable attribute of an individual” [7].

Ensuing articles reflect Kaplan’s exact concerns and suggestions, such as “authors often do not define race and ethnicity, have no rationale for including them, and use variable terminology” [9, 15]. Further, when presenting findings of racial or ethnic difference, authors generally “did not provide explanations of the difference” [11], with researchers still confused by the difference between race and ethnicity [12, 13] and a lack of transparency in the methods used to assess both concepts [13].

C.L. Ford, a practitioner of Public Health Critical Race Praxis, points out that lack of context for race in research leads to an erroneous understanding of it as the cause of health outcomes [16]. Figure 1 lists the racially responsive recommendations that were central themes supported by the majority of the papers and reflect a remarkable level of consensus. Appendix Table 1 further highlights the nuances of these recommendations with more details using sample statements from the manuscripts.

In addition, there have been recent editorials [2, 17, 18] and statements from biomedical journals and professional societies that highlight the issue of structural racism and to affirm their commitment to mitigate these issues [19–22]. Though racism in biomedical research is not their primary focus, many of these publications reinforce important themes identified in literature. For example, eliminating words and phrases that reflect systemic biases [19, 20], highlighting racism as a social determinant of health [2, 18], avoiding patient-blaming and “obfuscating the role of racism” [18], and ensuring that racial categories align with updated preferences [2, 23]. These narratives highlight the important role medical journals can play in increasing inclusion of studies that examine the role of structural racism and ensuring research findings are communicated appropriately [2, 24]. They add to the increasing calls to address the diversity among authors, reviewers, and editors, including a proposed role of an editor for diversity, equity, and inclusion [2, 19–22].

## Phase 2: Operationalize consensus recommendations through a novel framework and assessment questions

For a nearly three-decade span of literature, the degree of agreement in recommendations for standardizing the reporting of race in the US is strikingly high. We set out to develop a framework that captures the multiple dimensions of race encompassed in these recommendations.

**Fig. 2:**
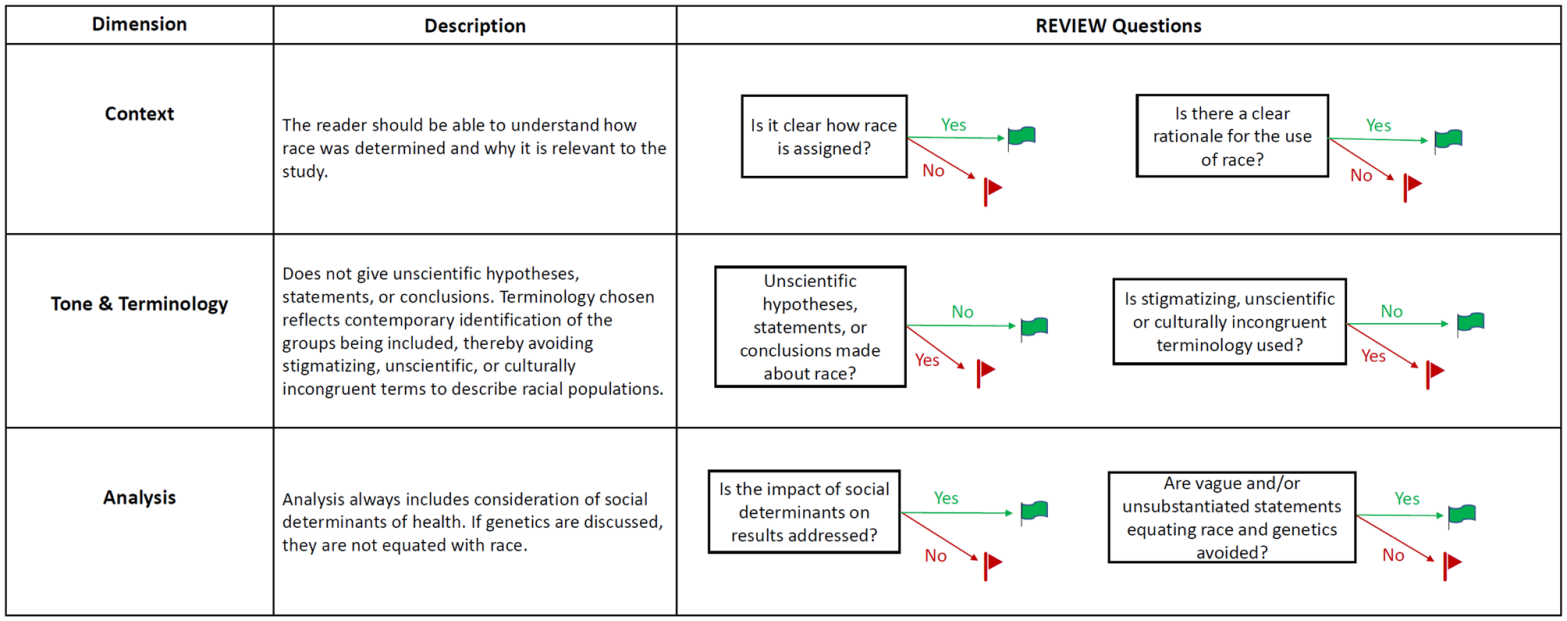
Racially Responsive Framework for US Biomedical Research. Figure 2 Dimensions of race included are 1) Context, 2) Tone & Terminology, and 3) Analysis. After a brief description of each dimension, the questions that form the Race Equity Vetting Instrument for Editorial Workflow (REVIEW) tool are presented

To guide editorial decisions on manuscripts that include race as a variable, or otherwise address race in a biomedical context, assessment questions are included in Fig. 2. These questions form the Race Equity Vetting Instrument for Editorial Workflow (REVIEW) tool.

### Dimension 1: Context

The context of race in biomedical research establishes how and why race is integrated into a study. How race is assigned is the first assessment question, which assists in reproducibility and comparability between studies. Although a straightforward starting point, this deceptively simple question generates a considerable amount of confusion and debate. The multiple layers involved include who assigns race (the individual, a healthcare worker, medical examiner, etc.), and what options the assigners have to choose from (how many races/levels of specificity, can multiple races be chosen, etc.). At this early stage of creating the REVIEW tool, it is clear that the methods for assigning race will need significant work to fully operationalize this first assessment question.

Since reporting race in biomedical research is not standardized, listing the rationale or clarifying the reason for the use of this imperfect tool in biomedical research is important. The earliest article identified in our structured literature search expresses this point that health inequalities and public health outcomes disproportionately affect minority populations. Thus, at the very least, authors should state that race is included in their research to “identify difference in health status amongst racial and ethnic minorities” [6]. More recently, Ioanndis et al. argue that as one of multiple other variables, a study may use race to further explore or identify issues with health inequities and systemic racism, capture influence on health status or ameliorate existing inequalities [3].

Of note, we chose not to include ethnicity in the framework or the assessment questions. This was a difficult decision, as ethnicity has the potential to be informative as a distinct category from race. However, race and ethnicity in the biomedical research literature are too often conflated or joined as terms with unspecified operational definitions (e.g., “race/ethnicity”) [11, 25]. This methodologically questionable practice poses a challenge when identifying the ethnicity of research subjects.

For example, Grafova and Jarrin [26] reported that Medicare administrative data on beneficiary ethnicity contained substantial misclassification errors. This finding was particularly problematic for Latino/Latina/Latinx/Hispanic (LHS +), Asian American/Pacific Islander, and American Indian/Alaska Native populations. In addition, many surveys forced choice option for single race or ethnicity. This second unwanted practice fails to allow for subgroup identity and poses challenges, e.g. affecting combined analyses between studies published in the US and in Latin America.

In summary, ethnicity is a complex concept that includes a blend of genetic, cultural identity, social and behavioral patterns [11]. In the US, ethnic identities may be interpreted dynamically to relate to country or region of origin, nativity, and generation [27], and thus may vary across research databases. Self-identification of ethnicity may be a fluid and ongoing process in the US that may attenuate or revitalize identities and groups [28]. To explicate ethnicity in the in-depth way it needs would require an additional layer of heterogeneity that is beyond the scope of this paper. Thus, we made the decision to have a singular focus on race.

### Dimension 2: Tone & terminology

Tone and terminology are the aspects of a biomedical paper where racial bias is most likely to be recognized. While building the framework, we discovered that tone was not included in the recommendations identified in the literature review. Thus, the following tone assessment question is designed to fill that critical gap: “Are unscientific hypotheses, statements or conclusions made about race?”.

Terminology choices, even at this early stage of creating an assessment tool, will clearly be one of the most difficult questions to operationalize. Three of the minority communities most commonly included in biomedical data each have multiple potential names. African American or Black? Latino, Latina, Latinx, Hispanic, or LHS + ? Native American, American Indian/Alaska Native, or Indigenous?

While consensus may be lacking on terminology choices for these US populations, the goal is clear. That is, the terminology chosen reflects contemporary identification of the groups being included, thereby avoiding stigmatizing, unscientific, or culturally incongruent terms to describe racial populations.

### Dimension 3: Analysis

The last dimension of race included in the REVIEW tool addresses whether the analysis of race data is presented in a comprehensive, scientifically responsible way. This dimension of race was especially difficult to define and operationalize. After extensive discussion, we concluded that social determinants of health (SDoH) should always be considered due to the substantial research in this field.

The final report of the World Health Organization Commission on SDoH provides perspective on the importance of the first screening question:

These inequities in health, avoidable health inequalities, arise because of the circumstances in which people grow, live, work, and age, and the systems put in place to deal with illness [29].

Additionally, the CDC just launched an initiative to address systemic racism as a public health threat [30].

Although it would be preferable if the studies themselves address SDoH, researchers could cite other articles that provide relevant data that SDoH could contribute to understanding their findings.

Another complex issue is how race and genetics should be handled in biomedical research. While researchers may have used these terms interchangeably in the remote past, this is being increasingly challenged with the advances in study of genes and technology in the past few decades. At a minimum, acknowledging the complexity of race and genetics is important, especially due to the “reductionist” tendency of biomedicine that may tempt us to seek a one factor-one disease approach. In the face of such uncertainty, we felt it is fair that authors explore differences in a candid manner and do not make vague, unsubstantiated statements equating genetics and race.

From the literature search, we found one explanation for much of the confusion of a genetic and/or biologic basis of race. The publication by a CDC-led workgroup in 1993 points out that much of the methods for collecting race data is influenced by the OMB, starting with a 1977 directive to consistently gather data on white, black and other [6]. Unfortunately, although clearly stated by OMB that their guidelines have no scientific basis, it seems that the consistent reporting had the opposite effect by creating the illusion of a biomedical meaning for race.

The authors of the 1993 CDC article clearly articulate a more nuanced approach to race and genetics/biology: “…while race may have some biological basis, its significance is mainly derived from social arrangements. Thus, race should be viewed within public health surveillance as a sociological phenomenon. Race and ethnicity are not risk factors – they are markers used to better understand risk factors” [6]. This statement is as true today as when it was written almost three decades ago.

## Phase 3: Pilot test the review tool

**Fig. 3:**
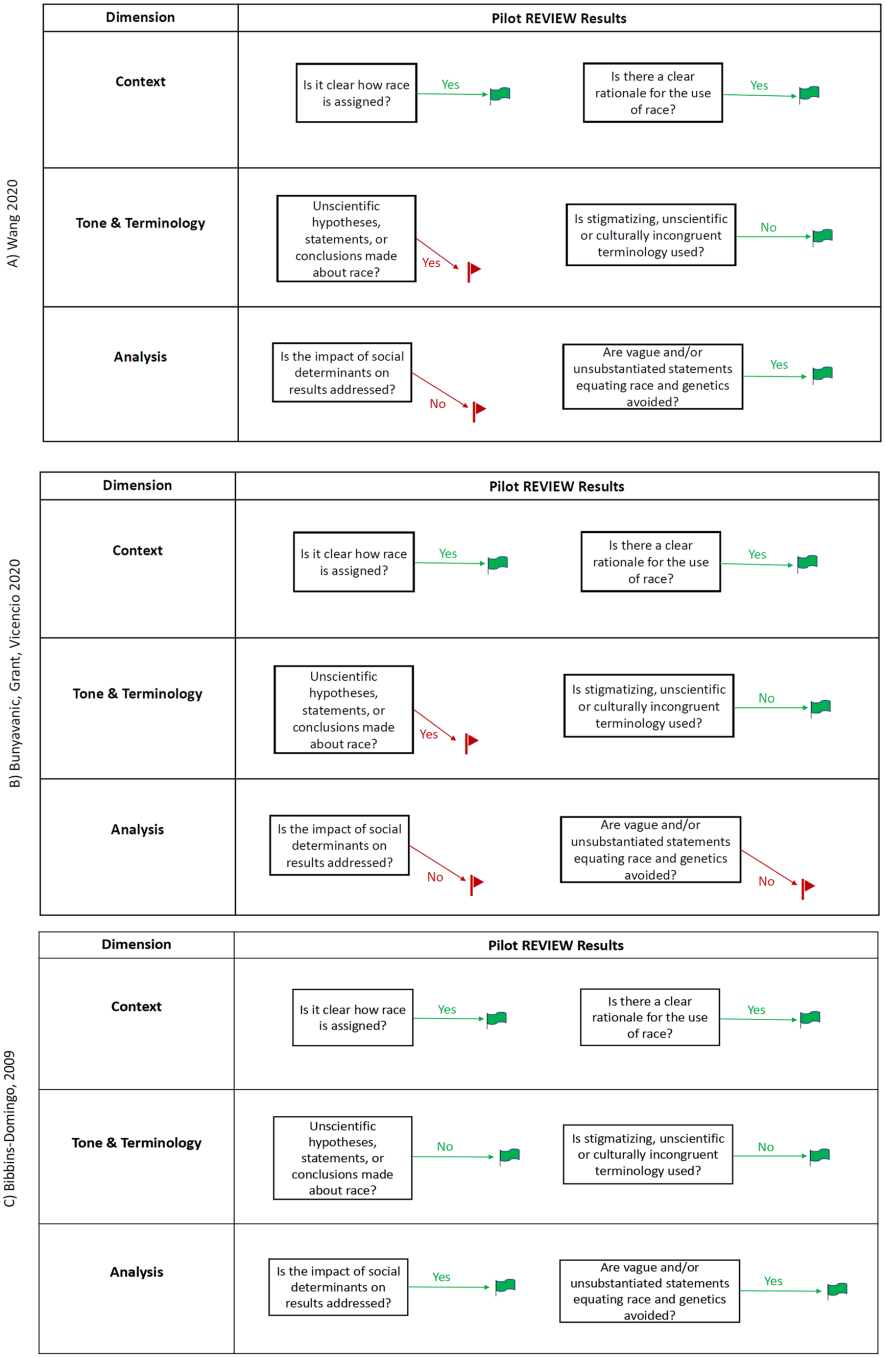
Pilot Testing the REVIEW Tool. Figure 3 The REVIEW tool as applied to three articles, two of which received considerable post-publication concerns for racial bias: **A**) Wang 2020 [25], **B**) Bunyavanic, Grant, Vicencio 2020 [31], and **C**) Bibbins-Domingo et al. 2009 [32]. Using these assessment questions, the two controversial papers were flagged for racial bias concerns by the REVIEW tool

In the pilot testing phase, we applied the REVIEW tool to three articles. Two of the articles garnered significant post-publication attention for racial bias, while the third is an exemplary article of a balanced approach to race in research. Figure 3 shows the results of pilot testing the REVIEW tool.

The first paper in pilot testing, Wang 2020, is a “white paper” on race and ethnicity in the cardiology workforce published in the *Journal of the American Heart Association* [25]. This work was widely criticized as overtly racist [33] and subsequently retracted by the journal with a commitment to investigate how the paper came to be published [34, 35]. Figure 3A demonstrates that applying the screening tool to this manuscript results in flagging concerns in Dimension 2 (Tone & Terminology) and Dimension 3 (Analysis).

The second paper in pilot testing is a research letter that appeared in the *Journal of the American Medical Association* [31]. The authors hypothesized that the disproportionately high infection and death rates due to COVID-19 in Blacks was due to genetic racial differences in density of nasal angiotensin-converting enzyme (ACE) 2 receptors. This paper has not been retracted. Media stories document concerns that the paper is inherently racist [34, 36], while others cite the fallacy of their theory due to low incidence of the receptor in question for the Latino community, which has also experienced high rates of COVID-19 [1]. The REVIEW tool flagged this publication on both Dimensions 2 and 3 in pilot testing (Fig. 3B).

The third article in the pilot testing phase, Bibbins-Domingo et al. 2009, published in the *New England Journal of Medicine*, is an example of a balanced approach to race in biomedical research [32]. The authors acknowledge a lack of understanding of risk factors for heart failure in young adults. Their longitudinal study addressing potential effects of clinical factors and social determinants of health in the outcomes for the mostly African-American study population.

Our Phase 3 pilot testing demonstrates that the REVIEW tool would have raised multi-dimensional concerns in both controversial papers. For the third article with a balanced approach to race cleared the pilot testing with no red flags. These results show that the REVIEW tool could have assisted editors to identifying manuscripts with troubling racial bias concerns in the pre-production editorial process.

## Next steps

To fully operationalize the REVIEW tool, the next step is to test this tool on a large sample of biomedical articles to determine if it is useful across the full range of racial bias, not just at the extremes.

An additional step is a nationwide initiative to work through the areas where consensus is lacking on race. How race is defined, terminology choices, and the role of genetics are areas that will benefit from efforts to build consensus on the national level. To reiterate an earlier point, an international focus would be preferable, however, the complexity of how race is addressed even within the US makes this line of research challenging. We actively encourage researchers in other countries to consider adopting the REVIEW tool to their needs.

## Conclusion

Using established research methods, we searched the literature for recommendations, created a framework to capture the relevant dimensions of race, and demonstrated successful pilot testing of the REVIEW assessment tool for biomedical research publications.

Additional steps will be needed to fully realize the potential of the REVIEW tool. Yet, even at this early stage, the REVIEW tool demonstrates the ability to prevent the publication of racially biased biomedical articles. With a standardized assessment tool, data on the handling of race and racial bias can be analyzed in the biomedical literature.

## Supplementary information

Below is the link to the electronic supplementary material.

Supplementary file1 (PDF 120 KB)
