# Supplementary material for: Feasibility of an Assessment Tool as a Data-Driven Approach to Reducing Racial Bias in Biomedical Publications
Source: J Med Syst. 2021 Dec 17;46(1):10. doi: 10.1007/s10916-021-01777-w (PMC8682034; doi:10.1007/s10916-021-01777-w)
Supplement: Supplementary file 1 — Supplementary file1 (PDF 120 KB) [file 10916_2021_1777_MOESM1_ESM.pdf]

Supplemental Table 1: Recommendations identified from literature review and sample quotes that highlight the problems with use of race in research

Recommendations to address problem:	Manuscript comments that highlight the problem:	
	**Sample A**	**Sample B**
**Reason for use of race as a variable specified**	"…key problems including: a) a failure from researchers to differentiate between the concepts of race and ethnicity where both concepts are often used interchangeably or merged into a single entity termed "race/ ethnicity"; b) an inappropriate use of racial categories to ascribe ethnicity; c) a lack of transparency in the methods used to assess both concepts; d) failure to address limits associated with the classification and use of racial or ethnic taxonomies and; e) failure to recognize the social meaning of race in discrimination and racism studies. (Moubarac 2013)"	"…it is possible for a biomedical journal to improve authors' description of how they measured race and ethnicity, but policy statements are not sufficient. Implementing changes… can improve such description, but ensuring that authors provide a hypothesis-driven rationale for reporting race/ethnicity is more difficult. (Winkler 2006)"
**How race was assigned is described**	"An explanation of who classified individuals as to race, ethnicity, or both, the classifications used, and whether the options were defined by the investigator or the participant should be included in the Methods section. The reasons that race/ethnicity was assessed in the study also should be described in the Methods section. Race/ethnicity of the study population should be reported in the Results section. (JAMA 2021)"	"there may be a tendency to both undertheorize race and ethnicity and overemphasize their importance. Given the long, troubled history of scientific racism, it is crucial that biomedical researchers carefully consider the theoretical significance of race or ethnicity for understanding health differences and to remain cautious against the potential for biological reductionism. (Lee 2013)"

**Race not used as a proxy for genetic variation**	“Race/ethnicity should not be used as a proxy for genetic variation.* Statements about genetic differences should be supported by evidence from gene studies. 17Genetic hypotheses should be firmly grounded in existing evidence, clearly stated, and rigorously tested. (Kaplan 2003)”	"A key concept to be addressed is the socially constructed aspects of the concepts of race and racial categories, as used in the USA, and the inappropriateness of using these kinds of categories in isolation for analyzing genetically based health issues. (Baer 2013)”
**Distinguished race/ethnicity as risk marker or factor**	In stating hypotheses and describing study results, authors should distinguish between race/ethnicity as a risk factor and race/ethnicity as a risk marker. (Kaplan 2003)”	"an appropriate beginning point for these discussions is an exploration of how human diversity is understood and classified, and what those classification systems mean and do not mean. Discussions should then consider what race and ethnicity are and are not. (Baer 2013)”
**Social Determinants of Health considered**	“In the interpretation of racial/ ethnic differences, all conceptually relevant factors should be considered, including racism and discrimination,*SES,† social class, personal or family wealth, environmental exposures, insurance status, age, diet and nutrition, health beliefs and practices, educational level, language spoken, religion, tribal affiliation, country of birth, parents’ country of birth, length of time in the country of residence, and place of residence. (Kaplan 2003)”	“…socioeconomic status such as income and education does not permit exploration of the complex attitudinal and behavioral factors that may have some association with skin color. (Winkler 2006)”

**Findings adjusted for Socio-Economic Status or label unadjusted findings**	"Because lack of adjustment for SES or social class is the most important potential source of bias in studies of racial/ ethnic differences, researchers should make every effort to adjust for conceptually relevant measures of SES or social class when comparing racial/ethnic groups.‡ Unadjusted findings should be clearly labeled as such, and in general they should be reported in conjunction with adjusted findings for comparison purposes. (Kaplan 2003)"	"...analyze related measures such as socioeconomic variables when available, and discuss the potential role of unmeasured confounders when reporting results by race/ethnicity. (Winkler 2006)"
**Race terms that are stigmatizing, unscientific, or immutable trait are avoided**	In describing racial/ethnic groups, authors should use terminology that is not stigmatizing, does not reflect unscientific classification systems, and does not imply that race/ethnicity is an inherent, immutable attribute of an individual. (Kaplan 2003)"	"...the terms used to describe race and ethnicity often have been inaccurate and inappropriate. The terms "Latino/ Hispanic,"3 "Asian,"4 and "white" or "Caucasian"5 have all been criticized for inaccuracy and ambiguity. The problem is compounded when observers such as researchers or clinicians classify individuals by race based on skin color and appearances, but even self-classification raises issues. (Winkler 2006)"
**Limitations of the methods used in categorizing race must be described as a caveat for understanding the results in the biomedical study.**	"…race and ethnicity are commonly reported variables in biomedical research, but how they were initially determined is often not described and the rationale for analyzing them is often not provided"520 , "When race is used as a variable, however, the task of researchers and medical editors is to ensure that the assessment of relevance is as accurate as possible, the criteria for categorizing race are described	"Third, even when researchers define race or ethnicity, they rarely provide information about how these variables are measured. Overall, fewer than one in five studies specified methods for determining participants' race or ethnicity" (p.31), "...commonly stated method for determining race or ethnicity was the use of preexisting records, such as birth certificates or medical records. These records, in turn, have well-known flaws regarding the validity and

precisely, and the limitations of race as an explanatory variable are recognized. (Winkler 2006)”

reliability of data on race and ethnicity (Hahn 1992", "In particular, as other content analyses have shown, most health researchers (1) fail to define race and ethnicity, (2) conflate the two concepts, (3) neglect to identify how people were assigned to racial or ethnic categories, and (4) omit the reason for including race and ethnicity in the study. (Gravlee 2008)”

References

1. Moubarac JC. Persisting problems related to race and ethnicity in public health and epidemiology research. Rev Saude Publica. 2013;47(1):104-15. doi: 10.1590/s0034-89102013000100014.
2. Winker MA. Race and ethnicity in medical research: requirements meet reality. J Law Med Ethics. 2006;34(3):520-5, 480. doi: 10.1111/j.1748-720x.2006.00065.x.
3. JAMA Network. Instructions for Authors. Authorship and Disclosures. Available from: https://jamanetwork.com/journals/jama/pages/instructions-for-authors#SecAuthorshipandDisclosures
4. Lee C. "Race" and "ethnicity" in biomedical research: how do scientists construct and explain differences in health? Soc Sci Med. 2009;68(6):1183-90. doi: 10.1016/j.socscimed.2008.12.036.
5. Kaplan JB, Bennett T. Use of race and ethnicity in biomedical publication. JAMA : the journal of the American Medical Association. 2003;289(20):2709-16. doi: 10.1001/jama.289.20.2709.
6. Baer RD, Arteaga E, Dyer K, Eden A, Gross R, Helmy H, et al. Concepts of race and ethnicity among health researchers: patterns and implications. Ethn Health. 2013;18(2):211-25. doi: 10.1080/13557858.2012.713091.
7. Gravlee CC, Sweet E. Race, ethnicity, and racism in medical anthropology, 1977-2002. Med Anthropol Q. 2008;22(1):27-51. doi: 10.1111/j.1548-1387.2008.00002.x.
